# A qualitative study on the symptoms and psychological characteristics of young Hwa-Byung patients

**DOI:** 10.1192/j.eurpsy.2022.1399

**Published:** 2022-09-01

**Authors:** S.-A. Park, J. Kim, S. Lee, S.-W. Choi

**Affiliations:** Duksung Women’s University, Clinical Psychology Lab, Dobong-gu, Seoul, Republic of Korea

**Keywords:** Hwa-Byung, Qualitative study, cultural-related syndrome, Anger

## Abstract

**Introduction:**

Hwa-Byung is characterized by feelings of anger or resent about unreasonable social violence and trauma. Although Prevalence of Hwa-Byung is high in middle-aged, recently Hwa-Byung is occurred in young people. And it is suggested that young Hwa-Byung patients will show different patterns from middle-aged.

**Objectives:**

The purpose of present study was to qualitatively analyze young Hwa-Byung patients’ experiences of symptoms and psychological characteristics related to antecedent events of Hwa-Byung.

**Methods:**

10 women aged 20s were Interviewed using semi-structured questionnaires to in-depth study on their experiences related to Hwa-Byung. The interview data were analyzed using phenomenological approach in order to understand the essence of experiences. In particular, it was analyzed through five steps according to Giorgi (1985). First, by repeatedly reading the material, recurring themes were identified. Second, the meaning units were divided to capture important parts of participants’ statements. Third, similar meaning units were grouped together. Fourth, the determined meaning units were described in psychological term. Finally, research data were integrated and presented according to the described meaning units. Reliability between coders was higher than the minimum reliability coefficient.

**Results:**

In symptoms, two themes and seven sub-themes were emerged. In psychological characteristics, three components and five sub-themes were derived. In particular, the antecedent events of young Hwa-Byung patients were related to vertical social relationships, suggesting that Hwa-Byung need to be understood under social context which make them angry.

**Conclusions:**

Present study revealed the social context of Hwa-Byung by discussing the differences between young and middle-aged patients, and furthermore, differences between Hwa-Byung and depression, PTSD patients.

**Disclosure:**

No significant relationships.

